# LOCAT (low-dose computed tomography for appendicitis trial) comparing clinical outcomes following low- vs standard-dose computed tomography as the first-line imaging test in adolescents and young adults with suspected acute appendicitis: study protocol for a randomized controlled trial

**DOI:** 10.1186/1745-6215-15-28

**Published:** 2014-01-17

**Authors:** Soyeon Ahn

**Affiliations:** 1 Medical Research Collaborating Center, Seoul National University Bundang Hospital, 82, Gumi-ro 173beon-gil, Bundang-gu, Seongnam-si, Gyeonggi-do 463-707, Republic of Korea

**Keywords:** Appendicitis, Tomography, X-ray computed, Radiation

## Abstract

**Background:**

Computed tomography is widely used to diagnose acute appendicitis. Many adolescents and young adults are exposed to the associated radiation. A recent single-institution trial has reported promising results for low-dose computed tomography; however, this technique has not yet been widely adopted. LOCAT (low-dose computed tomography for appendicitis trial), a multi-institution randomized controlled non-inferiority trial, aims to compare low-dose computed tomography and standard-dose computed tomography as the first-line imaging tests for adolescents and young adults, and therefore to test the generalizability of the previous single-institution trial results.

**Methods/Design:**

Participants with suspected appendicitis are randomly assigned to either the low-dose group (with a typical effective dose of 2 mSv) or the standard-dose group (as used in normal practice at each participating site, typically 8 mSv). The primary end point is the negative appendectomy rate (the percentage of the number of uninflamed appendices that were removed among all non-incidental appendectomies), which is a consequence of false-positive diagnoses, with a non-inferiority margin of 4.5 percentage points. The key secondary end point is the appendiceal perforation rate, which is a consequence of delayed (or false-negative) diagnoses. Participant recruitment will be continued until the number of non-incidental appendectomies for each group exceeds 444. The total number of expected participants approximates 3,000, including those not undergoing appendectomy.

**Discussion:**

In addition to the study protocol, we elaborate on several challenging or potentially debatable components of the study design, including the broad eligibility criteria, choice of the primary end point, potential effect of using advanced imaging techniques on study results, determining and adjusting the radiation doses, ambiguities in reference standards, rationale for the non-inferiority margin, use of the intention-to-treat approach and difficulties in defining adverse events.

**Trial registration:**

ClinicalTrials.gov NCT01925014

## Background

Acute appendicitis is one of the most common indications for emergency abdominal surgery [1]. Computed tomography (CT) has assumed a paramount position in the disposition of adult patients with suspected appendicitis in the developed world, owing to its many advantages over other diagnostic tests including ultrasonography [2,3]. Studies conducted in Korea (Park JH on behalf of the LOCAT group: Diagnostic imaging utilization in cases of acute appendicitis: multi-center experience, unpublished) and the United States [4-6] have reported preoperative CT utilization rates ranging from 93% to 98% in patients undergoing appendectomy between 2007 and 2011. CT is highly accurate, readily available, rapid, easy to perform and interpret, and rarely affected by bowel gas, severe abdominal pain or extreme body habitus [7]. Despite a historical debate [8], a number of recent studies [5,6,9-12] have consistently shown that the increased use of CT coincides with a reduction in the negative appendectomy rate (NAR) without an increase in the appendiceal perforation rate (APR). NAR is the percentage of the number of negative appendectomies (removal of an uninflamed appendix) out of all non-incidental appendectomies [6,8-12]. NAR and APR are two important reciprocal measures of the quality of care, indicative of false-positive and delayed (false-negative) diagnoses, respectively. The routine use of CT for patients suspected of having appendicitis has also been reported to be cost-effective through prevention of delayed or inaccurate diagnoses [13].

There has been a surge in CT use for diagnosing appendicitis during the last decade in the United States [5,6,8-12], indicating that the threshold for the decision to use CT may have declined. Over 250,000 appendectomies are performed in the United States each year [1], while approximately 95,000 were performed in the Republic of Korea in 2011 [14]. The vast majority of these patients undergo CT examination preoperatively [5,6,9,11,12]. Moreover, there is an even greater number of patients who undergo CT and do not finally undergo appendectomy. Factors contributing to these trends include improved CT technology, widespread availability, favorable reimbursement and a general shift in the culture of medicine toward defensive medicine [15] and dependency on imaging tests [16].

Many patients with suspected appendicitis are children or young adults [1], for whom CT radiation is of particular concern [17]. Although debatable, there are increasing concerns that even a single typical abdomen CT examination may increase the risk of carcinogenesis [17-19]. While such risk induced by an individual CT scan would be minute, multiplication by the large number of exposures may imply the real occurrence of cancer. With a greater awareness of the carcinogenic risk [19,20], it may no longer be certain if the benefits of CT in diagnosing appendicitis clearly outweigh the risk associated with the radiation doses traditionally used. It should be noted that the traditional radiation doses have historically been determined without robust scientific basis [21], with large variations in practice across hospitals [22]. Furthermore, while there is no rationale for using an identical dose for young appendicitis patients and elderly patients with malignancies, attempts have rarely been made to properly differentiate the dose levels according to the application.

Results from several studies have suggested that reducing the radiation dose by 50% to 80% does not significantly impair the diagnosis of appendicitis [23-25], although the dose reduction decreases image quality. Recently, a single-institution randomized controlled trial [26] demonstrated the non-inferiority of low-dose (LD) CT, which used a quarter of the standard dose (SD), compared to SD CT with respect to NAR (3.5% vs 3.2%; 95% CI for the difference, -3.8 to 4.6 percentage points) for adolescents and young adults with suspected appendicitis. However, the study had a potentially important limitation. While appendicitis is a very common disease encountered across emergency departments worldwide, it remains uncertain if the results of that particular study can be generalized to other institutions that are less experienced in using LD CT. At the time of writing, the LD CT technique has not been widely accepted as the standard of practice in many institutions. We have therefore proposed a multi-institution trial with a similar study design to confirm the generalizability of the results of the previous single-institution study. In this article, we summarize the protocol of the study, LOCAT (low-dose CT for appendicitis trial).

### Study objectives

The primary objective of LOCAT is to determine whether LD CT is non-inferior to SD CT as the first-line imaging test in regard to NAR for adolescents and young adults. In addition, LOCAT aims to disseminate the use of the LD CT technique throughout the participating sites through the implementation of the study protocol.

## Methods/design

### General

Conducting LOCAT at a site requires the approval of the site’s Institutional Review Board. LOCAT will be conducted in accordance with the Korean Good Clinical Practice Guideline [27]. Informed consent is a prerequisite. The protocol and the informed consent form should been approved by the ethics committee at each trial site (see Additional file 1).

**Figure 1:**
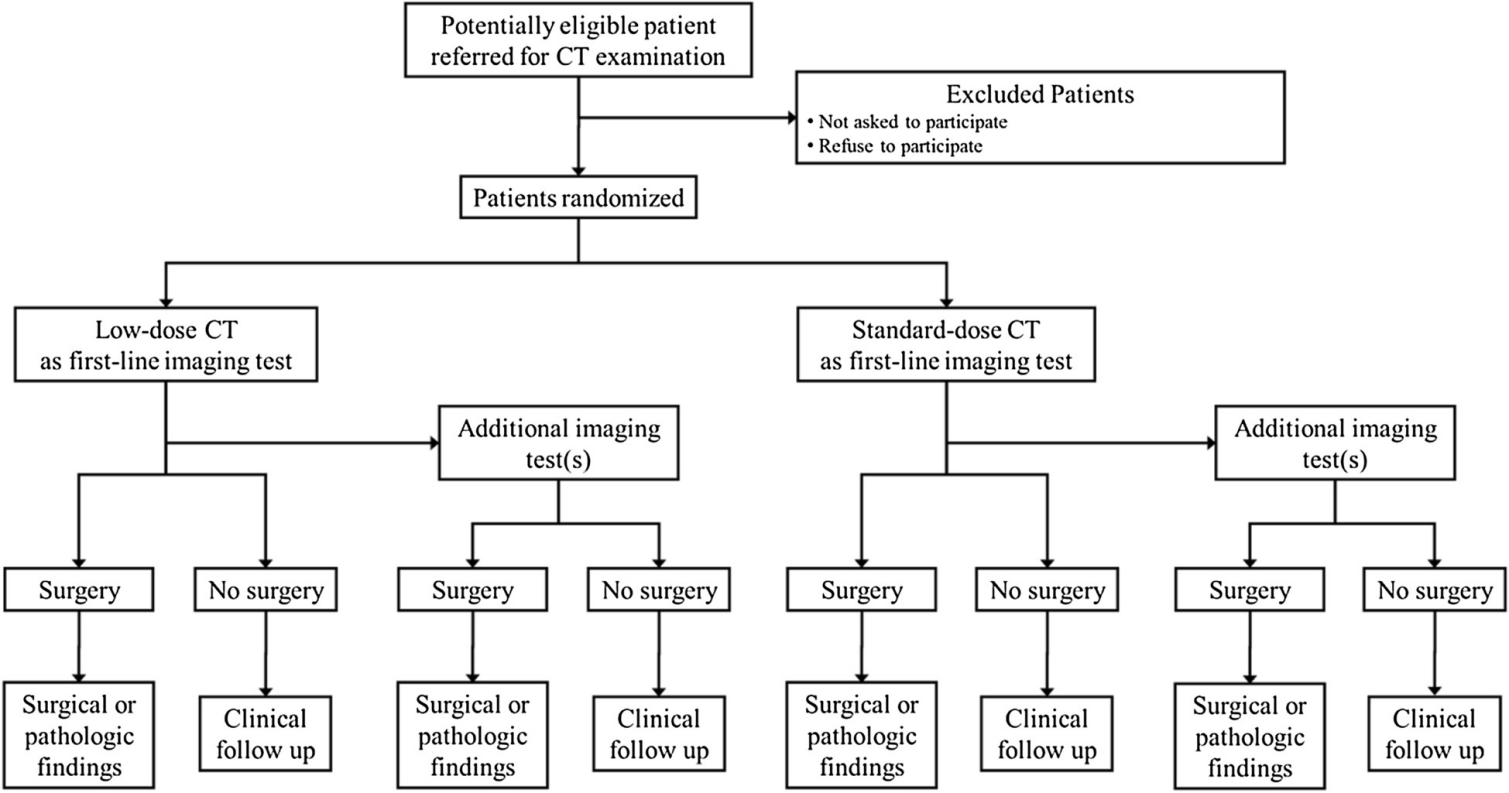
Study overview.

LOCAT is a multi-institution single-blind randomized controlled non-inferiority trial comparing LD CT (with a typical effective dose of 2 mSv) and SD CT (as used in normal practice at each site) as the first-line imaging test for adolescents and young adults (Figure 1). The primary end point is the negative appendectomy rate and the key secondary end point is the appendiceal perforation rate. Participating radiologists are members of the Korean Society of Abdominal Radiology. LOCAT Group members are listed in Additional file 2. LOCAT was commissioned by the Korean Ministry of Health and Welfare in 2013, which is partly funding the study. Recruitment has commenced in December 2013.

### Eligibility criteria

Eligible patients are aged 15 to 44 years of age, visiting emergency departments with suspected symptoms and signs of acute appendicitis, undergoing intravenous contrast-enhanced CT examination requested due to suspicion of appendicitis, and without any prior cross-sectional imaging tests to evaluate the presenting symptoms and signs. Patients with a slender body shape, prior history of allergy to iodinated intravenous contrast materials or prior history of renal insufficiency will generally be recommended to undergo ultrasonography instead of CT, and therefore, are unlikely to be enrolled in LOCAT. However, these are not absolute exclusion criteria. To reproduce normal practice at each site, the clinical suspicion of appendicitis as well as the need for a CT examination will be left to the discretion of the emergency department physicians on duty.

The eligibility criteria differ from those in previous studies measuring the effect of preoperative CT on NAR [5,6,8-12] in that LOCAT participants are limited to adolescents and young adults for whom the long-term risks of cumulative radiation are more relevant [17].

### Site recruitment

The LOCAT office has been regularly sending out invitation letters to all members of the Korean Society of Abdominal Radiology (a nationwide society of abdominal radiologists), which has 303 members from 119 hospitals as of September 2013. As site recruitment is primarily based on the voluntary participation of radiologist investigators, the study’s generalizability may be limited. Before the first participant at a site is registered, the site must complete a checklist and rehearse the study procedures. The rehearsal includes all relevant study procedures, except that the rehearsal patients will undergo SD CT regardless of the results of the random assignment (sham randomization). Patients participating in the rehearsal will not be included in the sample size or final analyses.

### Randomization

Participants who give informed consent will be randomly assigned to either LD or SD CT at a 1:1 ratio. Details of stratification and blocks are confidential. Sequentially numbered opaque sealed envelopes containing computer-generated random assignments will be prepared for each site. Randomization will take place at the time of the CT examination. To enter a participant into LOCAT, the radiology technologist on duty will open the next consecutively numbered envelope.

While it is not possible to blind the medical staff to the allocation because of obvious differences in image texture (dependent on the CT radiation dose), the participants and independent outcome assessors will be kept blinded to the allocation.

### Diagnostic interventions

Single breath-hold intravenous contrast-enhanced helical scans are obtained during the portal venous phase using 16- or higher detector-row CT machines. The imaging parameters are listed in Table 1. An iterative reconstruction is strongly recommended particularly for LD CT. Otherwise, there is no restriction in regard to scanner type or scan parameters, and the imaging protocol should follow normal practice at each site. For each CT scanner, the radiation dose should be adjusted to give a predefined target dose-length product (DLP) for an average-size patient [28] for LD and SD CT. The actual radiation dose should be recorded, as it is automatically modulated according to the individual’s body size and shape [29].

**Table 1:** CT imaging parameters

	**Imaging protocol**
Intravenous contrast enhancement	
Intravenous access	Antecubital, not lower extremity
Contrast material	Iodine, 400 to 800 mg/kg
Scan timing	Portal venous phase
Scan	
Range	From 4 cm above the liver dome to 1 cm below the ischial tuberosity
Collimation	Use all detector rows available
Automatic exposure control	Use all techniques available
Reconstruction	
Thick transverse images	Section thickness, 3 to 5 mm; overlap, 20% or more
Thin transverse images	Section thickness ≤2 mm; reconstruction interval ≤1 mm

The risk of cancer associated with the CT radiation for each group can be estimated using a method used in the previous single-institution trial [26]. According to the calculation in that trial, using SD CT (approximately 8 mSv) instead of LD CT (approximately 2 mSv) for 2,000 male or 1,800 female 30-year-old patients would result in one additional cancer.

#### LD CT

The target DLP is set at 130 mGy · cm, which corresponds to an effective dose of 2 mSv with a conversion factor of 0.015 mSv · mGy^–1^ · cm^–1^[30]. This ‘low’ dose was empirically determined based on experience in depicting the appendix using reduced tube currents [24,25,31]. It was subsequently used in the previous single-institution trial [26].

#### SD CT

For the SD group, the target DLP is left to the discretion of the site principal investigator (PI) in line with the normal practice at each site. The target DLP is typically 530 mGy · cm corresponding to 8 mSv, but should not exceed the typical dose used in normal practice at the site. In comparison, reference values often quoted at the time this article was written range from 7 to 10 mSv [32-34]. It should be noted that the site PI is allowed to change the target DLP for each CT machine for the SD group during the study period and that no lower limit is defined for the target DLP. This is because the “standard” dose used at each site may decrease gradually during the study period [35], as will be discussed later.

### CT image interpretation and radiologists

In daily clinical practice, site radiologists produce some CT reports by reviewing images using the multiplanar sliding slab averaging technique, a real-time image post-processing technique [36-38], which is widely used to review large thin-section CT datasets efficiently. Typically, the initial CT reports are made by attending radiologists during the daytime and by on-call radiologists after hours. The CT reports are produced using a predefined structured format. The likelihood of appendicitis is rated on a five-point Likert scale.

LD CT is less straightforward to interpret than SD CT due to the lower image quality. Furthermore, most radiologists have limited experience in using LD CT prior to joining LOCAT. Therefore, to ensure the safety of the participants, it is mandatory at a site that more than 80% of the radiologists potentially involved in CT interpretation should complete a self-learning course before the first participant at the site is registered. The training materials include introductory PowerPoint slideshows and LD CT cases selected from the previous single-institution study [26] with direct feedback for the appendix’s location and the final diagnosis.

### Additional imaging

If the diagnosis of appendicitis remains undetermined after the initial CT examination and clinical observation, then additional abdominal imaging test(s), including abdominal ultrasonography [39,40] or SD CT, can be performed at the discretion of the emergency department physician or surgeon. An additional imaging test is defined as one that is performed within 7 days of the initial CT to diagnose or rule out appendicitis.

### Primary end point

The primary end point is NAR. As a secondary analysis, an alternative definition of NAR is used, which excludes cases with appendiceal neoplasms without superimposed appendicitis, as appendectomy would be indicated for such patients (Park JH on behalf of the LOCAT group: Diagnostic imaging utilization in cases of acute appendicitis: multi-center experience, unpublished). Any surgery performed for the treatment of presumed appendicitis is counted as non-incidental appendectomy, even though the surgical procedures may be more extensive than simple appendectomy (for example, ileocecectomy).

### Important secondary end points

#### Clinical outcomes

• APR, defined as the percentage of the number of perforated appendicitis for all confirmed appendicitis cases [8-11]

• The proportion of patients requiring additional imaging test(s) to diagnose or rule out appendicitis

• Delay in patient disposition

 ➢ Interval from CT acquisition to appendectomy in patients undergoing non-incidental appendectomy

 ➢ Interval from CT acquisition to hospital discharge in patients not undergoing surgery

• Length of hospital stay associated with non-incidental appendectomy, defined as the interval from CT acquisition to hospital discharge after non-incidental appendectomy

#### Radiologic outcomes for the diagnosis of appendicitis

• Diagnostic performance

 ➢ Area under receiver-operating-characteristic curve

• Diagnostic confidence

 ➢ Likelihood score for appendicitis in patients confirmed as having appendicitis

 ➢ Likelihood score for appendicitis in patients confirmed as not having appendicitis

### Reference standards

For participants undergoing abdominal surgery, the final diagnosis is made using the surgical and pathologic findings. Pathologic examinations of surgical specimens are performed by site pathologists during daily practice [41]. For participants not undergoing surgery, the outcome assessors will determine the final diagnosis based on the medical records and a standardized telephone interview conducted 3 months after the initial presentation. The outcome assessors are emergency department physicians or radiologists blinded to the allocation.

The histopathologic diagnosis of acute appendicitis is defined as neutrophil infiltration of the appendiceal wall [41]. If neutrophilic collections are confined to the mucosa, the diagnosis of acute appendicitis is based on the presence of mucosal ulcerations [42]. A diagnosis of appendiceal perforation is based on spillage of the appendiceal contents, peritonitis or an abscess observed during surgery [43], or a pathologically confirmed appendiceal wall defect due to transmural necrosis.

### Sample size

We assume 4% NAR following SD CT based on previous data from some of the sites [24,26,44]. The same NAR is assumed following LD CT. We judge 8.5% NAR to be clinically acceptable following LD CT, which corresponds to a non-inferiority margin of 4.5 percentage points, considering the potential reduction in carcinogenic risk associated with CT. With these assumptions and a 10% dropout rate, 444 non-incidental appendectomies per group are needed to obtain 90% statistical power with two-sided α = 0.05.

It should be recognized that participants not undergoing appendectomy are also included in LOCAT, although the required sample size was determined in terms of the number of appendectomies. Given that appendectomy is eventually performed in 40% to 44% of the patients undergoing appendiceal CT [24-26], we assume that appendectomy will be eventually performed in at least 30% of all LOCAT participants, considering the variability across sites. With this assumption, the total number of participants included in LOCAT is approximately 3,000.

If two or more life-threatening or fatal [45] serious adverse events (SAEs) are reported in any of the two groups, then LOCAT will be suspended and the Coordinating Committee will investigate if the events are attributable to study procedures and determine whether or not LOCAT should be terminated early.

### Analysis

All participants undergoing randomization will be included in the analysis in the groups to which they are originally assigned. While the intention-to-treat analysis will be used primarily, an additional per-protocol analysis will be performed. The NARs for both groups and the two-sided 95% CI for the differences will be calculated. The non-inferiority of LD CT compared to SD CT will be accepted if the upper bound of the two-sided 95% CI lies below the non-inferiority margin, which is 4.5 percentage points.

A similar non-inferiority analysis will be performed for APR with a non-inferiority margin of 10.0 percentage points. Chi-square tests, Fisher’s exact tests, Mann–Whitney U tests and receiver-operating-characteristic analysis (non-parametric Wilcoxon statistic) will be used to compare the other secondary end points. A two-sided *P* < 0.05 indicates statistical significance. If the study results show considerable variation across sites, generalized estimating equations will be used to account for the clustering effect by site.

## Discussion

Here we elaborate on several challenging or potentially debatable components of the LOCAT design, which we believe will be useful to other investigators designing or implementing a trial on the diagnosis of appendicitis.

### Broad eligibility criteria

LOCAT uses broad eligibility criteria to reflect normal practice at the sites and presumably in many other institutions where physicians maintain a reasonably sensitive standpoint in raising a clinical suspicion of appendicitis and then use imaging tests to confirm or rule out appendicitis.

First, the clinical suspicion for appendicitis will be left to the discretion of the emergency department physicians on duty. While a clinical suspicion for appendicitis is generally raised by known symptoms and signs including right lower quadrant pain, migration of pain, vomiting, tenderness and/or rigidity [46], we have chosen not to list any specific clinical criteria for defining the ‘suspicion for appendicitis’ in LOCAT. In general, patients with appendicitis have diverse presentations [47-49] and clinical assessments unavoidably require many physicians with different expertise, due to the high prevalence of the disease. Therefore, the adoption of any fixed clinical criteria may compromise the generalizability of the study findings. Wagner *et al*. [46] reviewed ten previous ‘high-quality’ studies and found that all of the studies used inclusion criteria of ‘suspected appendicitis’ or ‘abdominal pain’ without further definition. This is understandable, as fixing clinical criteria to investigate diagnostic clinical features is prone to circular logic.

Second, the need for a CT examination will also be left to the discretion of the emergency physicians on duty. While it may be debatable in Western countries [50] whether patients with typical presentations of appendicitis require a preoperative imaging test or not, such patients mostly undergo a CT examination in Korea where right-sided colonic diverticulitis, which often clinically mimics typical appendicitis [51,52], is a common alternative diagnosis [26]. While the need for a CT examination should be individualized for each patient, the decision threshold to utilize CT as the initial imaging modality may not be uniform among sites or emergency department physicians [2,50]. Although previous investigators have tried to divide the indication for performing a CT examination into two thresholds of atypical versus all suspected (including both typical and atypical) patients, a meta-analysis [50] has suggested that the dichotomy of atypical versus typical presentations is merely theoretical. Because of the broad eligibility criteria in LOCAT, patients with either typical or atypical presentations can be enrolled [3,50].

### Negative appendectomy rate versus appendiceal perforation rate as the primary end point

The primary end point is NAR and the most important secondary end point is APR. This is because NAR and APR are the two most important established reciprocal measures of quality of care [53] in the diagnosis of appendicitis. They represent the consequences of false-positive and delayed diagnoses, respectively. An inverse relation likely exists between NAR and APR if the overall performance of a diagnostic system is maintained stably. It has been asserted that a certain level of NAR (which was up to 20% before the introduction of CT) is an appropriate index of management and that a failure to maintain this surgical threshold is an indication of insufficient surgical aggressiveness and of an excessive rate of delayed diagnosis.

If LOCAT can prove the non-inferiority of LD CT compared to SD CT in terms of APR as well as NAR, the LOCAT results will be more conclusive than only proving the non-inferiority of NAR, in establishing LD CT as the first-line imaging test. Nevertheless, we have decided not to include APR as a co-primary end point for a number of reasons. First, ambiguity exists in defining appendiceal perforation as will be discussed later, which may partly explain the wide variation in reported APRs in previous studies [39] and across the sites in a retrospective study (Park JH on behalf of the LOCAT group: Diagnostic imaging utilization in cases of acute appendicitis: multi-center experience, unpublished). Second, while NAR explicitly represents the clinical consequences of false-positive diagnoses, APR is not so directly linked with false-negative diagnoses, as APR is also affected by many other factors including disease severity at the time of presentation and non-medical factors that delay treatment [54]. Third, appendiceal perforation, especially in a mild form, does not always affect clinical outcomes. Therefore, we have selected a single primary end point, NAR, in LOCAT.

Nevertheless, non-inferiority testing will be performed for APR as well as NAR. While testing two different hypotheses simultaneously (one for NAR and the other for APR) generally requires the control of Type I errors, we will use a hierarchical approach that can be used to test ordered hypotheses without the need for an α adjustment [55]. This fixed-sequence testing will allow the non-inferiority hypothesis for APR to be tested only if the non-inferiority for NAR is established first.

### Advanced CT imaging techniques

There have been remarkable advances in CT technology over the last decade, including improved spatial resolution, higher signal-to-noise ratio, faster scanning, increased use of multiplanar images and the introduction of the sliding slab averaging technique. Therefore, for the same radiation dose, the CT imaging protocol in LOCAT is considered to be better for visualizing the appendix than the CT protocols employed in the previous studies, which measured the effect of preoperative CT on NAR [6,8-12].

For example, thin-section (≤2 mm) image datasets, in addition to conventional thick-sections (3 to 5 mm), should be available to the radiologists. These can be reviewed using the multiplanar sliding slab averaging technique. The two-tier (thick and thin) image reconstruction technique [56] and the sliding slab averaging technique [36-38] can enhance visualization of the appendix [24-26,31,57,58], potentially compensating for the lower quality of LD CT images. The importance of these techniques is often overlooked by radiologists, although many ordinary hospitals now have sufficient hardware and network resources to implement them.

### Radiation dose

CT is rapidly evolving. Because participant recruitment for LOCAT will take several years, sites are allowed to implement advances during the course of LOCAT so that CT imaging parameters remain as up to date as possible. Within the study protocol, changes to the imaging protocol, even including the radiation dose level (target DLP) for the SD group, are allowed during the study period.

There is considerable variation across the sites in the radiation doses conventionally used to diagnose appendicitis (Park JH on behalf of the LOCAT group: Diagnostic imaging utilization in cases of acute appendicitis: multi-center experience, unpublished). In addition, the standard-of-care radiation doses may gradually decrease to some extent during the study period, through advances in CT technology and greater awareness of the associated carcinogenic risk [35]. Taking into consideration these variations and changes, LOCAT has a unidirectional standpoint in determining and adjusting radiation doses: it is flexible regarding dose decreases in either group while being strictly against dose increases. For example, the principle for dose calibration is as follows. If the median DLP for a number of participants exceeds a predefined target by 10% then the dose adjustment is mandatory, while it is left to the discretion of the site PI whether or not to adjust for the reverse error. It should be noted that lowering the SD during the course of LOCAT will affect the study results toward the non-inferiority. Nevertheless, this policy is in line with the ultimate goal of the LOCAT Group, which is to disseminate the use of the LD CT technique throughout the sites and other hospitals over the course of LOCAT.

### Potential ambiguities in reference standards

The pathologic diagnosis of appendicitis is not always straightforward when inflammation is confined to the mucosa. A systematic review [47] found that the pathologic criteria for appendicitis were missing or inconsistent in many previous studies. To ensure diagnostic reproducibility across the pathologists and sites, we have prepared a guideline.

The definition of appendiceal perforation is also unclear in previous studies addressing APR [8-11,59,60]. Importantly, in the previous studies it is particularly unclear whether appendiceal perforation refers only to a gross periappendiceal abscess or generalized peritonitis, or also includes microscopic perforation with localized peritonitis of minimal extent. While the former can alter the patient’s prognosis or the treatment plan, the latter is unlikely to do so. In LOCAT, the aforementioned broad definition is used to cover both extreme types.

### Rationale for the non-inferiority margin

To justify the non-inferiority margin of 4.5 percentage points for NAR, we have summarized the previously reported NARs. Several previous studies found that there was a decrease in NAR from a range of 12% to 29% down to 3% to 11% with the introduction of preoperative CT, as reviewed by Coursey *et al*. [6]. According to more recent studies [5,6,11,26,60], which likely used modern CT scanners with radiation doses presumably similar to the SD in LOCAT, the reported NAR ranged from 3.0% to 8.2%. To our knowledge, there has been no randomized controlled trial demonstrating the efficacy or effectiveness of SD CT over a placebo (no CT), which could be used as the basis for statistical reasoning of the non-inferiority margin [61]. Instead, a meta-analysis [62] suggested a NAR of 16.7% without preoperative CT compared to 8.6% with preoperative CT. More recent large observational studies [4] reported NARs of 8.5% to 12% in patients who underwent preoperative ultrasonography instead of CT.

For the non-inferiority test for APR, we assume 25% APR following SD CT based on previous data (ranging from 23% to 31%) from some of the sites [24-26]. The same APR is assumed following LD CT. We judge 35% APR to be clinically acceptable following LD CT, which corresponds to a non-inferiority margin of 10 percentage points. With these assumptions and with the 10% dropout rate and 4% NAR, the sample size determined to conclude the non-inferiority in NAR, 444 non-incidental appendectomies per group, would have a power of 89% in concluding the non-inferiority in APR.

### Intention-to-treat versus per-protocol analysis

Although a per-protocol analysis is generally preferred in a non-inferiority trial due to the possibility that non-adherence can bias the study results toward non-inferiority [63], we have chosen to use the intention-to-treat analysis primarily for the following reason. The motivation behind LOCAT is to replace SD CT with LD CT as the first-line imaging test. In other words, LOCAT is intended to compare the two diagnostic pathways including each physician’s clinical assessment and final clinical judgment based on the integration of all available diagnostic information such as the additional imaging test results as well as the initial CT results. This comparison is different from comparing LD and SD CT in a simple test-to-test manner.

### Difficulties in defining adverse events

LOCAT is a diagnostic trial and there are difficulties in adopting the adverse event reporting policy conventionally used in general therapeutic trials. Since participants with various abdominal diseases will be enrolled into LOCAT, there is expected to be a significant diversity of events in the course of the many downstream diagnostic and therapeutic pathways following the initial CT examinations. In contrast, since virtually all diagnostic and therapeutic procedures in LOCAT, except for the LD CT technique, follow normal practice at each site, clinically and scientifically meaningful events are anticipated to be few in number and minor. Therefore, reportable events in LOCAT are limited to unexpected SAEs, so that the reporting can be meaningful and feasible.

Since participants with various diseases will be enrolled, it is inevitable that expectedness is defined broadly as being consistent with the natural course of management of a participant with a given suspected or established diagnosis. Judgments should be made on a sound medical and scientific basis, assuming the best treatment in the absence of co-morbidities. For example, if a final diagnosis of colitis is established during the course of treatment to explain an initial presentation in a participant, the diagnosis of colitis should be regarded as expected, and, therefore, not reportable.

The terms defining SAEs will be reserved for situations in which the adverse event truly fits the definition. For example, hospitalization is not reportable when it is for diagnostic or elective surgical procedures for a pre-existing condition and the outcome is uneventful (for example, an uneventful negative appendectomy). In contrast, if abdominal pain persists after the first hospital discharge and leads to another hospitalization in which appendectomy is performed, the event should be regarded as reportable. In LOCAT, a hospital stay of over 7 days following non-incidental appendectomy is regarded as a prolongation of hospitalization.

## Trial status

Recruitment commenced in December 2013.

## Abbreviations

APR: appendiceal perforation rate; CI: confidence interval; CT: computed tomography; DLP: dose-length product; LD: low-dose; LOCAT: low-dose CT for appendicitis trial; NAR: negative appendectomy rate; PI: principal investigator; SAE: serious adverse event; SD: standard dose.

## Competing interests

None of the LOCAT investigators including SA declare any competing interests.

## Authors’ information

The LOCAT Group is a voluntary collaborative research group of radiologists, physicians, surgeons, pathologists and scientists. The goals of the LOCAT Group are to develop, validate, disseminate and standardize safe, accurate, convenient and cost-effective diagnostic systems for acute appendicitis and other acute abdominal diseases.

## Supplementary Material

Additional file 1 List of ethics committees and status of approval (as of 14 January 2014). Click here for file

Additional file 2: **List of LOCAT investigators and their contributions to LOCAT**. Click here for file
