# Supplementary material for: LOCAT (low-dose computed tomography for appendicitis trial) comparing clinical outcomes following low- vs standard-dose computed tomography as the first-line imaging test in adolescents and young adults with suspected acute appendicitis: study protocol for a randomized controlled trial
Source: Trials. 2014 Jan 17;15:28. doi: 10.1186/1745-6215-15-28 (PMC3903028; doi:10.1186/1745-6215-15-28)
Supplement: Additional file 1 — List of ethics committees and status of approval (as of 14 January 2014). [file 1745-6215-15-28-S1.pdf]

List of Ethics Committees and Status of Approval

As of Jan 14 2014, the status of approval in each investigating site is as follows.

- Ajou University Hospital; approval is pending (AJIRB-DEV_DEO-13-231)
- Chung-Ang University Hospital; approved (C2013155 (1115))
- Daejin Medical Center, Bundang Jesaeng General Hospital; approved (DR13-03)
- Hallym University Sacred Heart Hospital; approval is pending
- Hallym University Kangnam Sacred Heart Hospital; approval with conditions (2013-12-100)
- Kangbuk Samsung Medical Center; approval is pending
- Kangwon National University Hospital; approved (2013-12-012)
- Korea University Ansan Hospital; approval is pending
- Korea University Guro Hospital; approval with conditions (2013-11-0004)
- Seoul National University Bundang Hospital; approved (B-1103-123-008)
- Soonchunhyang University Bucheon Hospital; approved (2013-10-001)
- Soonchunhyang University Hospital; approval with conditions (2013-009)

The LOCAT Group January 14, 2014
