# Supplementary material for: LOCAT (low-dose computed tomography for appendicitis trial) comparing clinical outcomes following low- vs standard-dose computed tomography as the first-line imaging test in adolescents and young adults with suspected acute appendicitis: study protocol for a randomized controlled trial
Source: Trials. 2014 Jan 17;15:28. doi: 10.1186/1745-6215-15-28 (PMC3903028; doi:10.1186/1745-6215-15-28)
Supplement: Additional file 2: — List of LOCAT investigators and their contributions to LOCAT. [file 1745-6215-15-28-S2.pdf]

The LOCAT Group

LOCAT Office Staff

Jae Yeon Heo, Cho Hee Kim, Yousun Ko, Ji Eun Lee, Min Jung Lee, Ri Young Na, Eun Jung Shin

Study conception, organization, funding, study design, site accrual, and manuscript drafting

Kyoung Ho Lee (Seoul National University Bundang Hospital)

Participant/site accrual

Kyuseok Kim (Seoul National University Bundang Hospital)

Site accrual

Chang Hee Lee (Korea University Guro Hospital)

Study design and manuscript drafting

Soyeon Ahn (Seoul National University Bundang Hospital)

Ji Hoon Park (Seoul National University Bundang Hospital)

CT radiation dose calibration

Yong Hwan Chung (Seoul National University Bundang Hospital)

Bon Seung Gu (Seoul National University Bundang Hospital)

Radiologist training program

Jea Min Cho (Seoul National University)

Bohyoung Kim (Seoul National University Bundang Hospital)

Min Hee Lee (Soonchunhyang University Bucheon Hospital)

Hyun Sik Woo (SMG-SNU Boramae Medical Center)

Hyun Kyung Yang (Seoul National University Bundang Hospital)

CT report quality control

Min-Jeong Kim (Hallym University Sacred Heart Hospital)

Pathologic examination quality control

Hye Seung Lee (Seoul National University Bundang Hospital)

Surgical management quality control

Sung-Bum Kang (Seoul National University Bundang Hospital)

Radiation hazard estimation

Kwang Pyo Kim (Kyung Hee University)

Insurance

Hyuk Jung Kim (Daejin Medical Center Bundang Jesaeng General Hospital)

Participating Sites

Ajou University Hospital: Young Chul Kim*

Chung-Ang University Hospital: Yoo Shin Choi, Mi Kyung Kim, Sung Eun Kim, Sung Bin Park[*]

Daejin Medical Center, Bundang Jesaeng General Hospital: Young Rock Ha, Suk Ki Jang, Hyuk Jung Kim[*]

Hallym University Kangnam Sacred Heart Hospital: Ji Young Woo[*]

Hallym University Sacred Heart Hospital: Min-Jeong Kim[*]

Kangbuk Samsung Medical Center: Pil Cho Choi, Sang Kuk Han, Mi Sung Kim[*], Heon-Ju Kwon, Dong Hyuk Shin

Kangwon National University Hospital: Seong Whi Cho[*], Chul Woo Park

Korea University Ansan Hospital: Sang Hoon Cha, Han Jin Cho[*], Suk Keu Yeom

Korea University Guro Hospital: Sung Hyuk Choi, Chang Hee Lee, Jongmee Lee[*], Yang Shin Park

Seoul National University Bundang Hospital: Soyeon Ahn, Yong Hwan Chung, Bon Seung Gu, Sung-Bum Kang, Bohyoung Kim, Joong Hee Kim, Kyuseok Kim, Young Hoon Kim, Hye Seung Lee, Jae Hyuk Lee, Kyoung Ho Lee, Yoon Jin Lee, Chan Jong Park, Ji Hoon Park[*], Hyun Kyung Yang

Soonchunhyang University Bucheon Hospital: Ho Jung Kim, Hae Kyung Lee, Min Hee Lee[*], Boem Ha Yi

Soonchunhyang University Hospital: Seong Sook Hong[*]

[*]Site principal investigator.
